# 510 Antibiotic Serum Concentrations in Burn Patients with Augmented Renal Clearance and Adjusted Regimens

**DOI:** 10.1093/jbcr/irae036.145

**Published:** 2024-04-17

**Authors:** Scott W Mueller, Cameron Gibson, Arek J Wiktor

**Affiliations:** UCHealth and University of Colorado School of Pharmacy, Aurora, CO; University of Colorado, Aurora, CO; University of Colorado Hospital, Aurora, CO; UCHealth and University of Colorado School of Pharmacy, Aurora, CO; University of Colorado, Aurora, CO; University of Colorado Hospital, Aurora, CO; UCHealth and University of Colorado School of Pharmacy, Aurora, CO; University of Colorado, Aurora, CO; University of Colorado Hospital, Aurora, CO

## Abstract

**Introduction:**

Burn patients have altered pharmacokinetics, in part, due to a high incidence of augmented renal clearance (ARC). Augmented renal clearance is associated with subtherapeutic antibiotic concentrations necessitating dosing regimens that are greater than FDA approved doses. The objective of this cohort analysis was to assess antibiotic concentrations in patients with ARC specifically when real-time therapeutic drug monitoring was not available for the antibiotic.

**Methods:**

This retrospective descriptive cohort was performed at an ABA verified burn center. All patients with an antibiotic serum concentration measured between 2/2022 and 7/2023 and confirmed ARC were included. Patients were excluded if the only antibiotic concentration available was an aminoglycoside or vancomycin.

**Results:**

In all, 10 patients (90% male) had 15 non-real time antibiotic concentration assessments. The median (range) age, weight, TBSA, day since injury, serum creatinine and urine collection creatinine clearance corresponding to the drug concentration were 38 years (18-53), 85 kg (51-190), 24% (3-68.25), 13 days (6-32), 0.77 mg/dL (< 0.2-1.53), and 200 mL/min (96-336), respectively. The most common antibiotic concentration assessed was piperacillin (PIP, n=9), followed by meropenem (MER, n=3), cefepime (n=2), and linezolid (n=1). Piperacillin/tazobactam was administered as 22.5g/day continuous infusion and resulted in a median (range) PIP concentration of 43.7 mg/L (25-117). A single outlier of 117 mg/L occurred in a patient who was no longer in ARC, but reexhibited ARC days later and had a follow-up PIP concentration of 32 mg/L. Two MER concentrations representing a 2g every 8 hours using a 6 hour infusion were 13.9 mg/L at the end of infusion and 7.3 mg/L one hour after infusion. MER 1g every 8 hours with a 3 hour extended infusion resulted in a trough concentration of 1.2 mg/L. Cefepime 2g every 6 hours with a 3 hour extended infusion resulted in a peak and trough of 43.8 and 29.6 mg/L. Linezolid 600mg orally every 8 hours resulted in a trough of 4.21 mg/L. No adverse events were identified.

**Conclusions:**

Burn patients with ARC may require more aggressive dosing regimens to reach adequate antibiotic concentrations. A large prospective study is required to identify appropriate antibiotic dosing strategies to overcome pharmacokinetic alterations in this population, especially in the setting of pathogens with high minimum inhibitory concentrations.

**Applicability of Research to Practice:**

Although antibiotic target concentrations have not been established in the burn population, this cohort suggests antimicrobial regimens should be adjusted in burn patients with ARC. The lack of timely therapeutic drug monitoring for most antimicrobials may hamper patient specific dosing regimens to adequately treat infectious pathogens with elevated minimum inhibitory concentrations.

